# MLMDA: a machine learning approach to predict and validate MicroRNA–disease associations by integrating of heterogenous information sources

**DOI:** 10.1186/s12967-019-2009-x

**Published:** 2019-08-08

**Authors:** Kai Zheng, Zhu-Hong You, Lei Wang, Yong Zhou, Li-Ping Li, Zheng-Wei Li

**Affiliations:** 1 School of Computer Science and Technology, China University of Mining and Technology, Xuzhou, 221116 China; 2 Xinjiang Technical Institutes of Physics and Chemistry, Chinese Academy of Sciences, Ürümqi, 830011 China; 3 College of Information Science and Engineering, Zaozhuang University, Zaozhuang, 277100 China

**Keywords:** microRNA, Disease, Association prediction, Auto-encoder neural network, Random forest

## Abstract

**Background:**

Emerging evidences show that microRNA (miRNA) plays an important role in many human complex diseases. However, considering the inherent time-consuming and expensive of traditional in vitro experiments, more and more attention has been paid to the development of efficient and feasible computational methods to predict the potential associations between miRNA and disease.

**Methods:**

In this work, we present a machine learning-based model called MLMDA for predicting the association of miRNAs and diseases. More specifically, we first use the *k*-mer sparse matrix to extract miRNA sequence information, and combine it with miRNA functional similarity, disease semantic similarity and Gaussian interaction profile kernel similarity information. Then, more representative features are extracted from them through deep auto-encoder neural network (AE). Finally, the random forest classifier is used to effectively predict potential miRNA–disease associations.

**Results:**

The experimental results show that the MLMDA model achieves promising performance under fivefold cross validations with AUC values of 0.9172, which is higher than the methods using different classifiers or different feature combination methods mentioned in this paper. In addition, to further evaluate the prediction performance of MLMDA model, case studies are carried out with three *Human* complex diseases including *Lymphoma*, *Lung Neoplasm*, and *Esophageal Neoplasms*. As a result, 39, 37 and 36 out of the top 40 predicted miRNAs are confirmed by other miRNA–disease association databases.

**Conclusions:**

These prominent experimental results suggest that the MLMDA model could serve as a useful tool guiding the future experimental validation for those promising miRNA biomarker candidates. The source code and datasets explored in this work are available at http://220.171.34.3:81/.

## Background

MicroRNAs (miRNAs) are a large number of endogenous non-coding RNAs which transcribed as short hairpin precursors (~ 70 nt) [1, 2]. Recently, miRNA genes were discovered expressed in some types of diseases including Arthritis, Adenoid Cystic, Arteriosclerosis Obliterans, Immune Thrombocytopenic Purpura, and Idiopathic Pulmonary Hypertension exceptionally [3–10]. Therefore, more and more researchers believe that miRNAs could associate with sorts of disease. With the progression of biotechnology and accumulate of theories, a great quantity of miRNA–disease associations have been found and confirmed [11–14].

Although making use of the association between miRNAs and diseases could improve prognosis of the patients, the cost of confirming the relationship between miRNAs and diseases by experimental method is extremely high. Therefore, more and more computational methods have been developed in recent years [15–25]. Jiang et al. proposed a network-based approach to predict disease-miRNA associations [26]. Mork et al. built a model named miRPD which can definitely infer miRNA–protein-disease associations [27]. In order to further utilize miRNA-target interaction information, Xuan et al. built a prediction model named human disease-related miRNA Prediction (HDMP) according to weighted *k* most semblable node [28]. A prediction method named MIDP using random walk on the network was constructed by Xuan et al. [29]. This method reduced the negative impact of noisy data through restarting the walking. Chen et al. developed a prediction model named heterogeneous graph inference for miRNA–disease association prediction (HGIMDA) by mapping confirmed miRNA–disease associations into a heterogeneous graph [30]. Chen et al. developed regularized least squares for miRNA–disease association (RLSMDA) which can only use diseases without confirmed miRNAs to discover the association between diseases and miRNAs [31]. A model named ranking-based KNN for miRNA–disease association prediction (RKNNMDA) can predict unconfirmed miRNA without utilizing confirmed miRNAs, built by Chen et al. [32].

In this study, we propose a novel computational method, called MLMDA, based on the machine learning algorithm to predict miRNA–disease associations. MLMDA integrates different classes of information, including miRNA sequence information, disease semantic information, miRNA–disease association information and miRNA function information. An improvement to this approach is the introduction of sequence information to predict potential associations. Specifically, miRNA and disease similarity matrixes can be first computed respectively according to miRNA–disease association, the miRNA functional similarity and disease semantic similarity information. Second, MLMDA combines the matrixes of disease as a gathered similarity matrix. Third, auto-encoder is used to reduce the dimensionality of feature vectors for distinguishing miRNA–disease associations. Finally, the abstract feature is fed into random forest classifier to predict potential disease-related miRNA. For assessing the performance of MLMDA, we implement the fivefold cross validation method in the human microRNA disease database and get the AUCs of 91.72 ± 0.73%. Besides, to further evaluate the prediction performance of MLMDA model, three case studies are carried out with *Human* complex diseases including *Lymphoma*, *Lung Neoplasm*, *and Esophageal Neoplasms*. As a result, 97.5%, 92.5% and 90% of the top 40 predicted miRNAs are confirmed by two other miRNA–disease association databases, respectively. The above experimental results demonstrated that MLMDA is a powerful and efficacious method for predicting potential miRNA–disease associations.

## Results

### Performance evaluation 

#### Prediction of miRNA–disease association

**Fig. 1:**
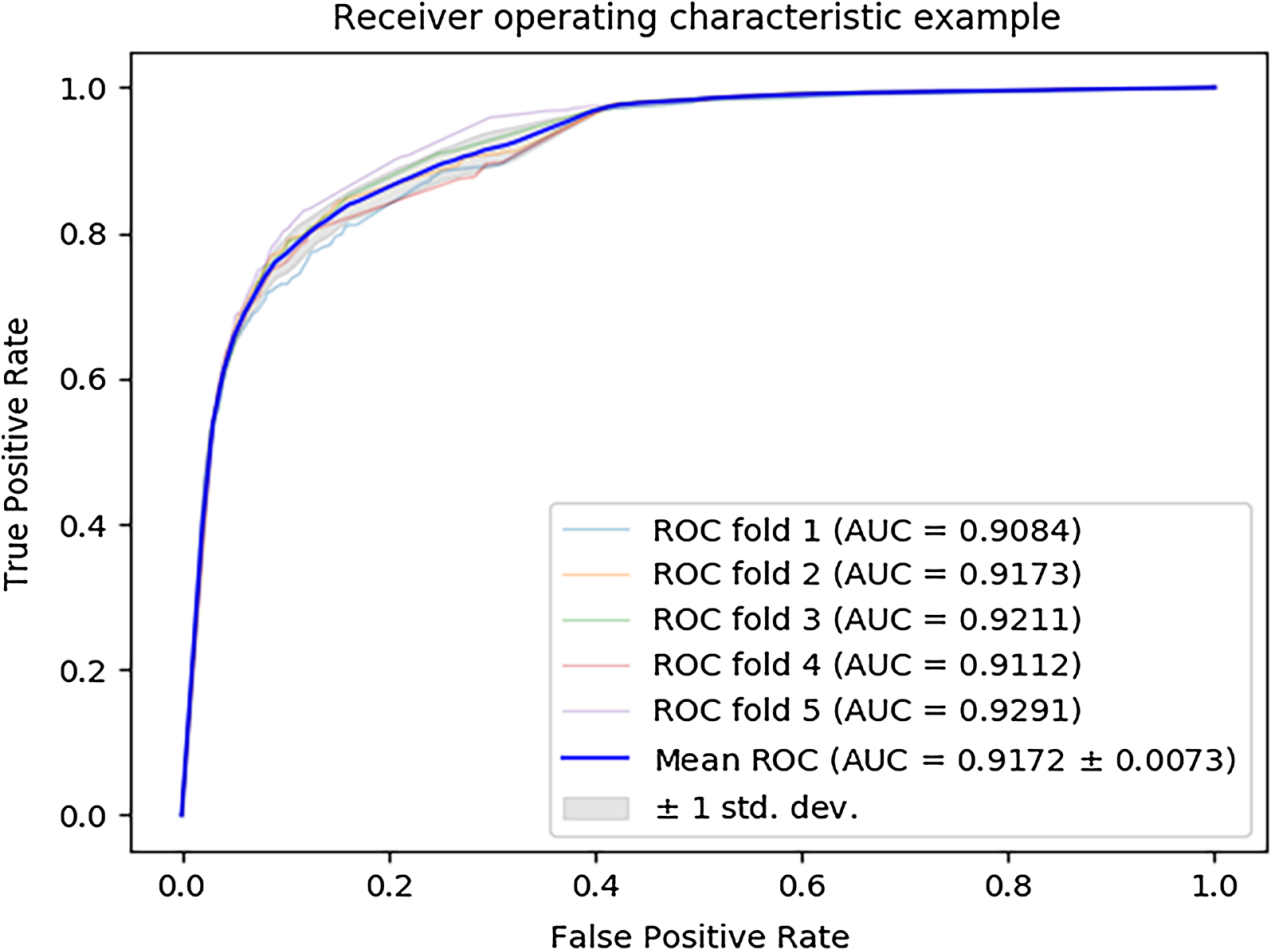
ROC curves performed by MLMDA on HMDD v3.0 dataset

We make use of fivefold cross validation according to the marked miRNA–disease associations in HMDD v3.0 to estimate the performance of MLMDA. The MLMDA gain a mean area under the receiver operation curve (AUC) of 91.72 ± 0.73% which is the average of AUCs of 90.84%, 91.73%, 92.11%, 91.12% and 92.91% in fivefold cross validation as showed in Fig. 1 and the yielded averages of accuracy, recall, precision and f1-score come to be 83.77%, 78.61%, 87.68% and 82.90% as showed in Table 1.

**Table 1:** Five-fold cross-validation results performed by MLMDA on HMDD v3.0 dataset

Testing set	Accuracy (%)	Recall (%)	Precision (%)	F1-score (%)
1	82.24	77.47	85.64	81.35
2	84.29	79.01	88.35	83.42
3	83.74	77.35	88.69	82.64
4	83.43	78.68	86.95	82.61
5	85.19	80.57	88.78	84.48
Average	83.77 ± 1.08	78.82 ± 1.31	87.68 ± 1.35	82.90 ± 1.15

#### Comparison with different classifier models

**Fig. 2:**
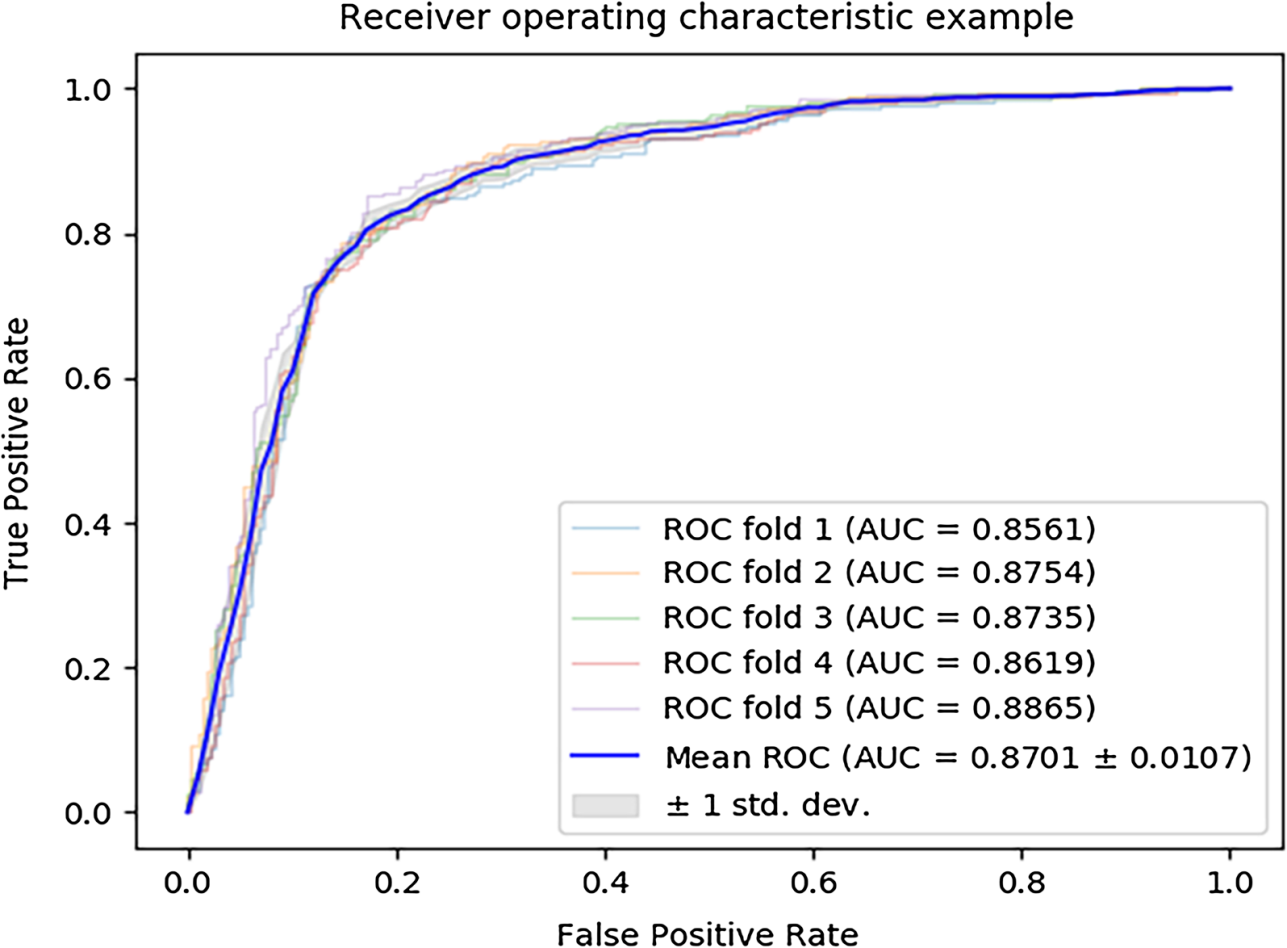
ROC curves performed by SVM model on HMDD v3.0 dataset

**Fig. 3:**
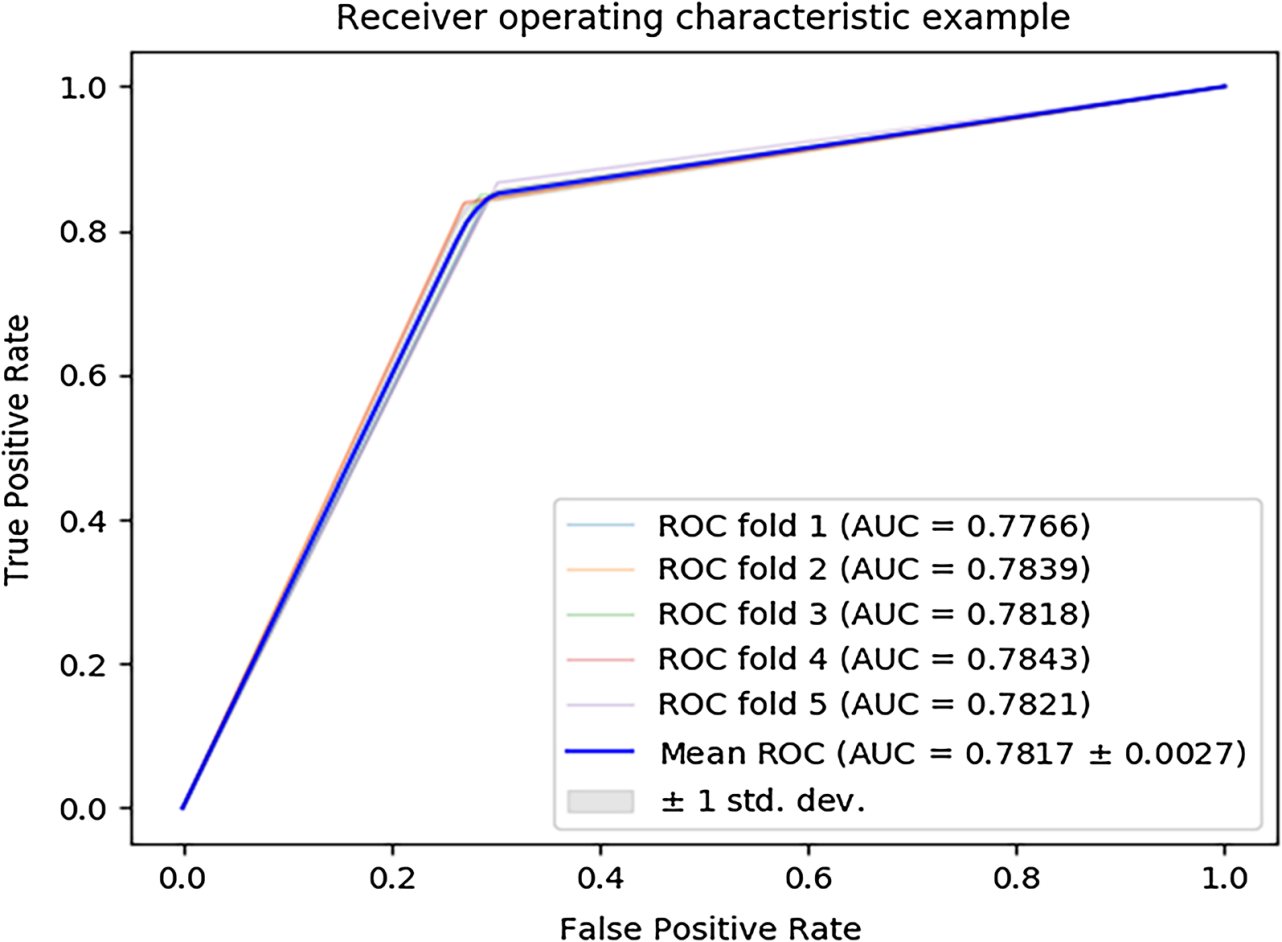
ROC curves performed by DT model on HMDD v3.0 dataset

In order to test the performance of MLMDA model using the Random Forest classifier, we compare it with different classifier models. Here, two models consisting of the state-of-the-art support vector machine (SVM) classifier and decision tree (DT) classifier are constructed to compare with the MLMDA model. In particular, all three models use the same training set and test set. In the experiment, SVM model achieves AUC of 87.01 ± 1.07% in the average of AUCs of 85.61%, 87.54%, 87.35%, 86.19% and 88.65% under fivefold cross validation, as shown in Fig. 2. Decision tree achieves AUC of 78.17 ± 0.27% in the average of AUCs of 77.66%, 78.39%, 78.18%, 78.43% and 78.21% under fivefold cross validation, as shown in Fig. 3. The yielded averages of accuracy, recall, precision and f1-score come to be 81.47%, 79.50%, 81.88% and 80.66% as show in Table 2 and 78.17%, 84.75%, 74.91% and 79.52% as in show Table 3. For a more intuitive comparison of performance, the evaluation parameters for the three models are summarized in Table 4. The experimental results show that MLMDA has achieved the best results among the evaluation criteria of accuracy, Precision, F1 and AUC. In summary, MLMDA has better performance and robustness than the other two models, especially in the accuracy, AUC and F1 values that can quantify the performance of the entire model, although MLMDA model is not as good as SVM model are in recall. Based on the above results, the random forest is the most suitable classifier for the model.

**Table 2:** Five-fold cross-validation results performed by SVM model on HMDD v3.0 dataset

Testing set	Accuracy (%)	Recall (%)	Precision (%)	F1-score (%)
1	81.00	76.54	83.04	79.66
2	81.59	79.84	81.86	80.83
3	81.20	79.01	81.70	80.33
4	81.20	80.66	80.66	80.66
5	82.39	81.48	82.16	81.82
Average	81.47 ± 0.55	79.50 ± 1.89	81.88 ± 0.85	80.66 ± 0.78

**Table 3:** Five-fold cross-validation results performed by DT model on HMDD v3.0 dataset

Testing set	Accuracy (%)	Recall (%)	Precision (%)	F1-score (%)
1	77.66	84.56	74.31	79.11
2	78.38	83.67	75.67	79.47
3	78.18	84.91	74.84	79.56
4	78.43	83.97	75.60	79.56
5	78.21	86.65	74.13	79.91
Average	78.17 ± 0.30	84.75 ± 1.16	74.91 ± 0.71	79.52 ± 0.28

**Table 4:** The comparison results of MLMDA model, SVM model and DT model on HMDD v3.0 dataset

Model	Accuracy (%)	Recall (%)	Precision (%)	F1-score (%)	AUC (%)
SVM	81.47	79.50	81.88	80.66	87.01
DT	78.17	84.75	74.91	79.52	78.17
MLMDA	*83.77*	78.82	*87.68*	*82.90*	*91.72*

MLMDA obtains the highest value in the evaluation criteria (italics)

#### Comparison with different feature descriptors

**Fig. 4:**
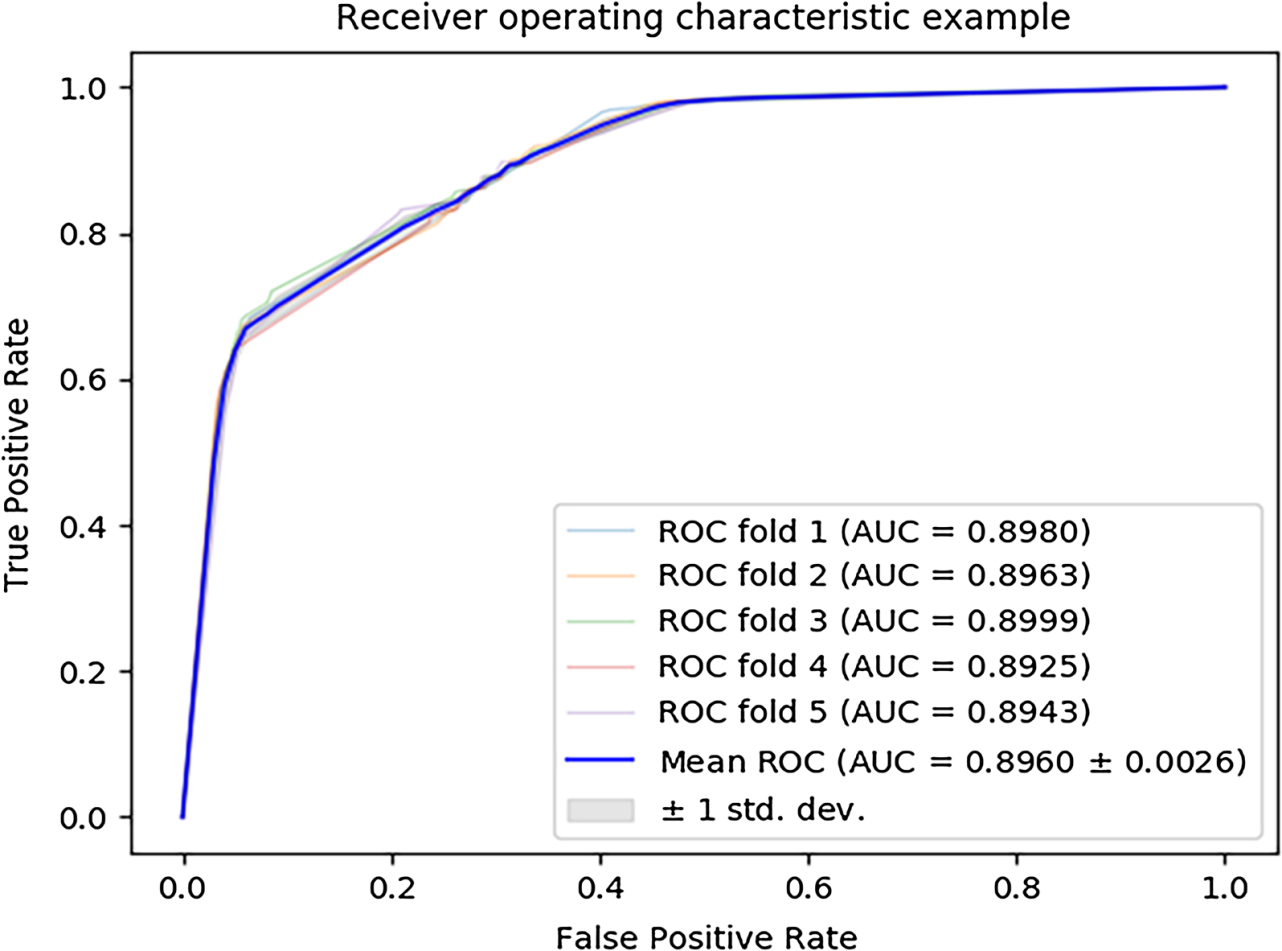
ROC curves performed by MLMDA_sim model on HMDD v3.0 dataset

**Fig. 5:**
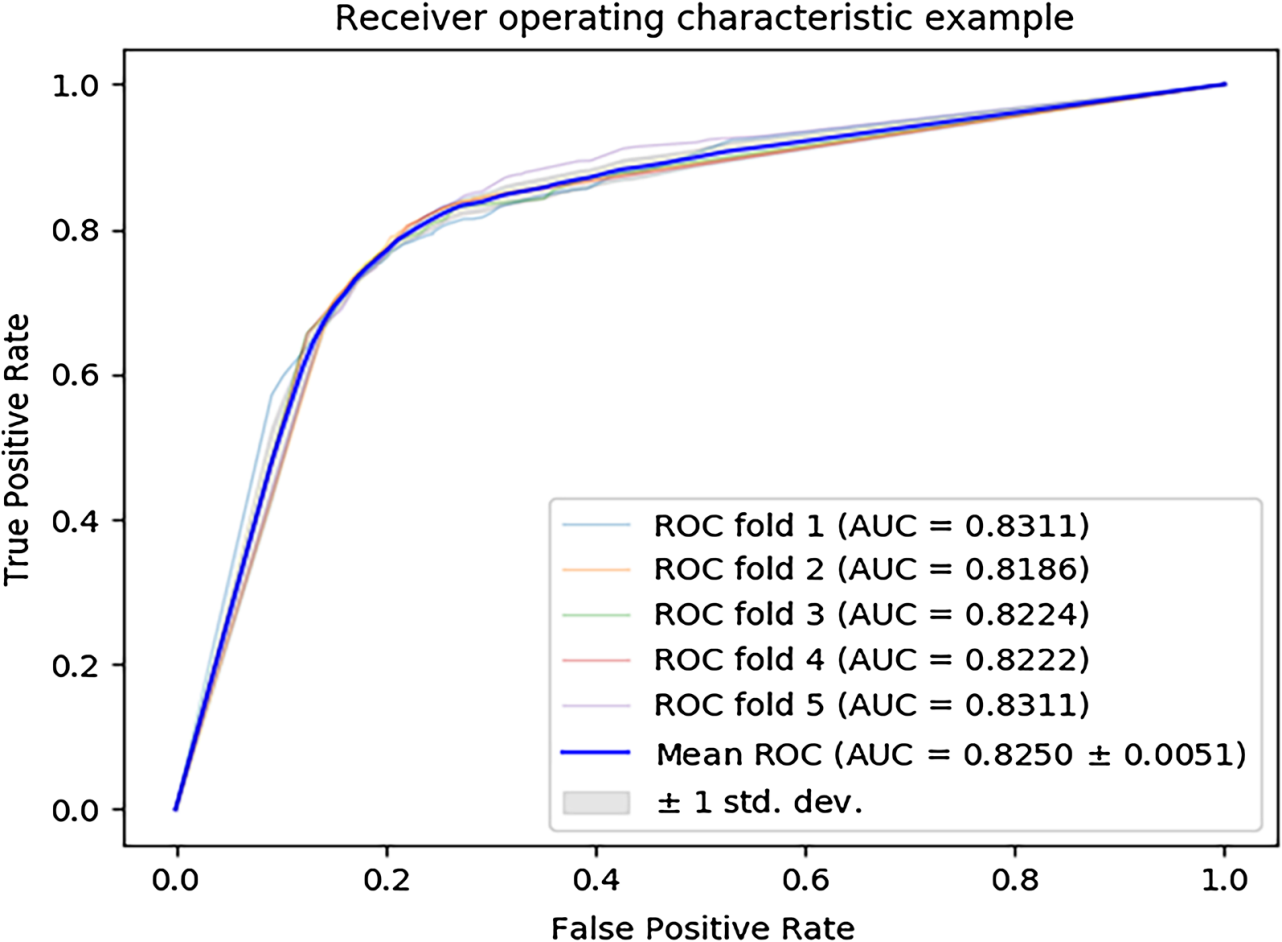
ROC curves performed by MLMDA_ds model on HMDD v3.0 dataset

In order to verify that the proposed descriptor represents the validity of the feature information, different descriptors are constructed to be compared to the proposed descriptor. In detail, the proposed descriptor MLMDA is composed of miRNA similarity information, disease similarity information and miRNA sequence information; the descriptor “MLMDA_ds” is composed of disease similarity information and miRNA sequence information; the descriptor “MLMDA_sim” is composed of disease similarity information and miRNA similarity information. The descriptor “MLMDA_sim” model gains a mean AUC of 89.69 ± 0.0026% which is the average of AUCs of 89.80%, 89.63%, 89.99%, 89.25% and 89.43% in fivefold cross validation (Fig. 4). The yielded averages of accuracy, sensitivity, precision and f1-score come to be 79.38%, 85.61%, 76.15% and 80.59% as show in Table 5. The descriptor “MLMDA_ds” model gets a mean AUC of 0.8250 ± 0.0051 which is the average of AUCs of 83.11%, 85.70%, 85.61%, 85.61% and 85.56% in fivefold cross validation (Fig. 5). The yielded averages of accuracy, recall, precision and f1-score come to be 78.58%, 78.30%, 78.76% and 78.51% as show in Table 6. It is noteworthy that the performances of AUCs in MLMDA were greater than that of the above experimental methods in fivefold cross validation, which shows that our method has obvious prediction performance. By comparing the combination methods of multi-source data, we find that introducing sequence information can improve the accuracy and AUC.

**Table 5:** The comparison results of MLMDA model and $F_{sim}$ feature model based on fivefold cross validation

Testing set	Accuracy (%)	Recall (%)	Precision (%)	F1-score (%)
1	79.81	83.73	77.66	80.58
2	79.19	85.92	75.74	80.51
3	79.42	87.81	75.20	81.01
4	79.03	84.82	76.03	80.18
5	79.45	85.80	76.13	80.68
Average	79.38 ± 0.29	85.61 ± 1.51	76.15 ± 0.91	80.59 ± 0.58

**Table 6:** The comparison results of MLMDA model and $SD\left( {d\left( a \right)} \right)$ with $F_{seq}$ feature model based on fivefold cross validation

Testing set	Accuracy (%)	Recall (%)	Precision (%)	F1-score (%)
1	78.07	77.59	78.35	77.97
2	78.59	76.17	80.05	78.06
3	78.57	77.89	78.98	78.43
4	79.15	80.45	78.42	79.42
5	78.52	79.42	78.02	78.71
Average	78.58 ± 0.38	78.30 ± 1.66	78.76 ± 0.79	78.51 ± 0.58

Table 7 summarizes the results of five cross-validations of three descriptors using random forest classifier on HMDD v3.0, namely MLMDA, MLMDA_sim, and MLMDA_ds. Our descriptors have achieved the best results in all evaluation criteria except recall, which indicates that the proposed descriptor can improve the prediction effect. In particular, adding feature information can also cause noise to affect predictive performance. Our descriptors improve the performance of the prediction model while adding information, indicating that the proposed descriptor is more suitable for our model than the other two.

**Table 7:** The comparison results of MLMDA model, descriptor MLMDA_ds model and descriptor MLMDA _sim model by Random forest classifier

Descriptor	Accuracy (%)	Recall (%)	Precision (%)	F1-score (%)	AUC (%)
MLMDA _ds	78.58	78.30	78.76	78.51	82.50
MLMDA _sim	79.38	85.61	76.15	80.59	89.60
MLMDA	*83.77*	78.82	*87.68*	*82.90*	*91.72*

MLMDA obtains the highest value in the evaluation criteria (italics)

#### Comparison with related works

To evaluate the effectiveness of our approach, we use the HMDD dataset to compare the performance of MLMDA with the 6 state-of-the-art methods which are BNPMDA, miRGOFS, MDHGI, DRMDA, SPM, LMTRDA and NNMDA, as shown in Table 8 [22, 33–37]. Since the version of HMDD used in the state-of-the-art methods is different, and some methods do not report detailed evaluation indicators, here we only compare the reported AUC values to verify the effectiveness of our method. As can be seen from Table 8, the proposed method is only 1.9% worse than the highest NNMDA of AUC, the second highest in all methods and 1.35% higher than the average AUC. This is due to the fact that sequence information can describe miRNAs more comprehensively and deeply, and can be used as an excellent source of knowledge for predicting potential miRNA–disease associations.

**Table 8:** The comparison results of MLMDA model and related works

Method	AUC (%)
BNPMDA	89.80
miRGOFS	87.70
MDHGI	87.94
DRMDA	91.56
SPM	91.40
LMTRDA	90.54
NNMDA	93.60
MLMDA	91.72

### Case studies

We prove the degree of MLMDA which could forecast potential miRNA–disease associations and confirm a high percentage of the possible disease-related miRNAs by carrying out three case studies. This means that MLMDA makes dependable predictions. *Lymphoma*, *Lung Neoplasm*, and *Esophageal Neoplasms* are chosen to construct the three cases studies and training samples for the method are constructed by miRNA–disease pairs from HMDD v3.0. Whereafter, we use the top 20 and 40 candidates as the prediction lists and validate in two notable miRNA–disease association databases dbDEMC v2.0 and miR2Database [38, 39]. There is no repeat of the training samples and the prediction lists, because of arranging and authenticating candidate miRNAs.

In the first case study, *Lymphoma* is chosen as the example and we predict Lymphomas-related miRNAs by MLMDA. *Lymphoma* is a cancer that begins in infection-fighting cells of the immune system, called lymphocytes [40, 41]. As a result, 20 out of the top 20 and 39 out of the top 40 potentially miRNAs which associate with *Lymphoma* are verified by either dbDEMC and miR2 disease or other experimental studies, shown as Table 9. A malignant tumor is usually diagnosed at advanced stage and has a poor prognosis named *Lung neoplasms*. It is selected as the second case study and we use MLMDA to predict the potential associated miRNAs by ranked 771 miRNAs according to predicted scores. The results are shown in Table 10, 18 out of the top 20 and 37 out of the top 40 predicted miRNAs are verified in the experimental data. We choose *Esophageal Neoplasms* as the third investigated disease [42–45]. *Esophageal* cancer is a malignant tumor, the most common type of which is esophageal squamous cell carcinoma and adenocarcinoma. As shown in Table 11, the predicted scores of the candidate miRNAs are ranked and 36 were verified in the first 40 potential miRNAs associated with *Esophageal Neoplasms*.

**Table 9:** Prediction of the top 40 predicted miRNAs associated with *Lymphoma* based on known associations in dbDEMC v2.0 and miR2Databas

miRNA	dbDEMC	miR2D	miRNA	dbDEMC	miR2D
hsa-mir-191	Confirmed	Unconfirmed	hsa-mir-1	Confirmed	Unconfirmed
hsa-mir-195	Confirmed	Unconfirmed	hsa-mir-206	Confirmed	Unconfirmed
hsa-mir-30a	Confirmed	Confirmed	hsa-let-7c	Confirmed	Confirmed
hsa-let-7a	Confirmed	Confirmed	hsa-mir-106a	Confirmed	Confirmed
hsa-mir-183	Confirmed	Unconfirmed	hsa-mir-146b	Confirmed	Unconfirmed
hsa-mir-101	Confirmed	Unconfirmed	hsa-mir-132	Confirmed	Unconfirmed
hsa-mir-141	Confirmed	Unconfirmed	hsa-mir-29a	Confirmed	Unconfirmed
hsa-mir-145	Confirmed	Confirmed	hsa-mir-181b	Confirmed	Unconfirmed
hsa-mir-34a	Confirmed	Unconfirmed	hsa-mir-378	Confirmed	Unconfirmed
hsa-mir-223	Confirmed	Unconfirmed	hsa-mir-151a	Confirmed	Unconfirmed
hsa-mir-451	Confirmed	Unconfirmed	hsa-mir-181c	Confirmed	Unconfirmed
hsa-let-7e	Confirmed	Confirmed	hsa-mir-574	Confirmed	Unconfirmed
hsa-mir-125b	Confirmed	Unconfirmed	hsa-mir-214	Confirmed	Unconfirmed
hsa-mir-99a	Confirmed	Confirmed	hsa-mir-106b	Confirmed	Unconfirmed
hsa-mir-24	Confirmed	Unconfirmed	hsa-mir-137	Confirmed	Unconfirmed
hsa-mir-144	Confirmed	Unconfirmed	hsa-mir-30c-2	Confirmed	Unconfirmed
hsa-mir-449a	Confirmed	Unconfirmed	hsa-mir-590	Confirmed	Unconfirmed
hsa-let-7i	Confirmed	Unconfirmed	hsa-mir-7	Confirmed	Unconfirmed
hsa-mir-34c	Confirmed	Unconfirmed	hsa-mir-30	Unconfirmed	Unconfirmed
hsa-let-7 g	Confirmed	Unconfirmed	hsa-mir-196b	Confirmed	Unconfirmed

**Table 10:** Prediction of the top 40 predicted miRNAs associated with *Lung Neoplasm* based on known associations in dbDEMC v2.0 and miR2Database

miRNA	dbDEMC	miR2D	miRNA	dbDEMC	miR2D
hsa-mir-320b-1	Confirmed	Unconfirmed	hsa-mir-449b	Confirmed	Unconfirmed
hsa-mir-1266	Confirmed	Unconfirmed	hsa-mir-128	Confirmed	Unconfirmed
hsa-mir-616	Confirmed	Unconfirmed	hsa-mir-19b-2	Confirmed	Unconfirmed
hsa-mir-1228	Confirmed	Unconfirmed	hsa-mir-190a	Confirmed	Unconfirmed
hsa-mir-1307	Confirmed	Unconfirmed	hsa-mir-190b	Confirmed	Unconfirmed
hsa-mir-573	Confirmed	Unconfirmed	hsa-mir-634	Unconfirmed	Unconfirmed
hsa-mir-376	Unconfirmed	Unconfirmed	hsa-mir-512-2	Confirmed	Unconfirmed
hsa-mir-2110	Confirmed	Unconfirmed	hsa-mir-369	Confirmed	Unconfirmed
hsa-mir-455	Confirmed	Unconfirmed	hsa-mir-320b-2	Confirmed	Unconfirmed
hsa-mir-646	Confirmed	Unconfirmed	hsa-mir-320c-1	Confirmed	Unconfirmed
hsa-mir-655	Confirmed	Unconfirmed	hsa-mir-193	Confirmed	Unconfirmed
hsa-mir-516a-2	Confirmed	Unconfirmed	hsa-mir-618	Confirmed	Unconfirmed
hsa-mir-526a-1	Unconfirmed	Unconfirmed	hsa-mir-320d-1	Confirmed	Unconfirmed
hsa-mir-133	Confirmed	Unconfirmed	hsa-mir-339	Confirmed	Confirmed
hsa-mir-526a-2	Unconfirmed	Unconfirmed	hsa-mir-576	Confirmed	Unconfirmed
hsa-mir-384	Unconfirmed	Unconfirmed	hsa-mir-106b	Confirmed	Unconfirmed
hsa-mir-544a	Confirmed	Unconfirmed	hsa-mir-492	Confirmed	Unconfirmed
hsa-mir-1285	Confirmed	Unconfirmed	hsa-mir-513c	Confirmed	Unconfirmed
hsa-mir-15b	Confirmed	Confirmed	hsa-mir-193b	Confirmed	Unconfirmed
hsa-mir-92a-2	Confirmed	Unconfirmed	hsa-mir-519c	Confirmed	Unconfirmed

**Table 11:** Prediction of the top 40 predicted miRNAs associated with *Esophageal Neoplasms* based on known associations in dbDEMC v2.0 and miR2Database

miRNA	dbDEMC	miR2D	miRNA	dbDEMC	miR2D
hsa-mir-204	Confirmed	Unconfirmed	hsa-mir-199a	Confirmed	Unconfirmed
hsa-mir-15b	Confirmed	Unconfirmed	hsa-mir-222	Confirmed	Unconfirmed
hsa-mir-224	Confirmed	Unconfirmed	hsa-mir-221	Confirmed	Unconfirmed
hsa-mir-335	Confirmed	Unconfirmed	hsa-mir-1-1	Unconfirmed	Unconfirmed
hsa-mir-138	Confirmed	Unconfirmed	hsa-mir-208	Unconfirmed	Unconfirmed
hsa-let-7 g	Confirmed	Unconfirmed	hsa-mir-191	Confirmed	Unconfirmed
hsa-let-7i	Confirmed	Unconfirmed	hsa-mir-328	Confirmed	Unconfirmed
hsa-mir-139	Confirmed	Unconfirmed	hsa-mir-200b	Unconfirmed	Unconfirmed
hsa-mir-140	Confirmed	Unconfirmed	hsa-mir-16-2	Confirmed	Unconfirmed
hsa-let-7	Confirmed	Unconfirmed	hsa-mir-186	Confirmed	Unconfirmed
hsa-mir-212	Unconfirmed	Unconfirmed	hsa-mir-1	Confirmed	Unconfirmed
hsa-mir-144	Confirmed	Unconfirmed	hsa-mir-20b	Confirmed	Unconfirmed
hsa-mir-499	Confirmed	Unconfirmed	hsa-mir-142	Confirmed	Unconfirmed
hsa-mir-124-1	Confirmed	Unconfirmed	hsa-mir-370	Confirmed	Unconfirmed
hsa-mir-96	Confirmed	Unconfirmed	hsa-mir-30a	Confirmed	Unconfirmed
hsa-mir-181b	Confirmed	Unconfirmed	hsa-mir-497	Confirmed	Unconfirmed
hsa-mir-16-1	Confirmed	Unconfirmed	hsa-mir-29b-2	Confirmed	Unconfirmed
hsa-mir-19b-1	Confirmed	Unconfirmed	hsa-mir-374a	Confirmed	Unconfirmed
hsa-mir-92-1	Confirmed	Unconfirmed	hsa-mir-432	Confirmed	Unconfirmed
hsa-mir-182	Confirmed	Unconfirmed	hsa-mir-320a	Confirmed	Unconfirmed

## Materials and methods

### Human miRNA–disease associations database

In the experiment, we use Human microRNA Disease Database (HMDD) established by Li et al. as the benchmark dataset [46], which can be downloaded at http://www.cuilab.cn/hmdd. This dataset includes 32,281 confirmed miRNA–disease pairs with 1102 miRNAs and 850 diseases. In pretreatment, we remove some pairs which cannot be confirmed by the miRBase. So, we choose all marked miRNA–disease associations that each miRNA can match its own sequence as positive set. Besides, the same amount of the unconfirmed miRNA–disease associations is selected as negative set. After screening, an adjacency matrix is established on this basis. The element ((),()) is assigned to 1, otherwise it is assigned to 0, if disease () and miRNA () are confirmed that they have a relationship in the HMDD v3.0 database [47].

### MiRNA functional similarity

The miRNA functional similarity information we use in the experiment was provided by Wang et al., which according to the assumption that miRNAs which have same function are more likely to relate with similar disease, vice versa [48–50]. The miRNA functional similarity information can be described as a matrix $FS$, which contains 495 rows and 495 columns. The element $FS\left( {m\left( a \right),m\left( b \right)} \right)$ of $FS$ represents the similarity value between miRNA $m\left( a \right)$ and miRNA $m\left( b \right)$. It can be downloaded from http://www.cuilab.cn/files/images/cuilab/misim.zip. This part of the data is only used in case studies.

### Disease semantic similarity

Medical Subject Headings (MeSH) diseases descriptors offer a strict system for classing disease and we use it to abstract disease semantic similarity. In this database, the nodes are diseases and the edges connecting two nodes from parent node to child node could describe a Directed Acyclic Graph (DAG) for each disease. In this work, the relations between miRNA-related diseases are constructed by disease MeSH descriptors. We download MeSH descriptors form the National Library of Medicine (http://www.nlm.nih.gov/). Disease ${\text{D}}$ can be described as ${\text{DAG}}_{d} = D, T_{d} , E_{d}$, where ${\text{T}}_{d}$ is a node set containing disease ${\text{D}}$ and its ancestor diseases $E_{d}$ is an edge set containing the corresponding edges [48]. Here, we use the previous method that according to MeSH diseases descriptors to compute disease semantic similarity [28]. Particularly, the semantic value of disease D is described as the effect of disease t, as follows:

1 $$\left\{ {\begin{array}{*{20}l} {D1_{d} \left( t \right) = 1} & \quad { if \,t = D} \\ {D1_{d} \left( t \right) = max\left\{ {\Delta *D_{d} \left( {t^{\prime}} \right)|t^{\prime} \in\,children\,of\,t} \right\}} & \quad {if\, t \ne D} \\ \end{array} } \right.$$

where $\Delta$ is the semantic contribution decay factor and if $t$ is unlike to $D$, it will cut down the contribution of disease $t$. On the contrary, the contribution of disease $D$ is equal to 1.

In addition, we define the semantic value $DV\left( D \right)$ as follows:

2 $$DV\left( D \right) = \mathop \sum \limits_{{t \in T_{d} }} D_{d} \left( t \right)$$

If disease $d\left( i \right)$ and $d\left( j \right)$ share larger part of their DAGs, two diseases will be more similar and their semantic similarity value could be computed based on this conjecture, defined as follows:

3 $$Sim1\left( {d\left( i \right),d\left( j \right)} \right) = \frac{{\mathop \sum \nolimits_{{t \in T_{d\left( i \right)} \cap T_{d\left( j \right)} }} \left( {D1_{d\left( i \right)} \left( t \right) + D1_{d\left( j \right)} \left( t \right)} \right)}}{{DV\left( {d\left( i \right)} \right) + DV\left( {d\left( j \right)} \right)}}$$

where $Sim1$ is a disease semantic similarity matrix. $Sim1\left( {d\left( i \right),d\left( j \right)} \right)$ is the semantic similarity of $d\left( i \right)$ and $d\left( j \right)$.

### Disease semantic similarity

We calculate disease semantic similarity with a diseases’ DAGs. They are built by MeSH descriptors novel edge-based method. On the whole, disease terms will have a larger contribution if they have higher specificity in semantic metric. Thus, preserving the characteristic of diseases is the key to the high precision of computation model. Firstly, we calculate the semantic characteristic of all diseases. We define a disease term $t$, its semantic characteristic is described as follows [51].

4 $$D2_{d} \left( t \right) = log\left( {1 + \frac{number \;of\;DAGs \;including\;t}{number\; of\; disease } } \right)$$

Secondly, calculating the semantic similarity value between disease $d\left( i \right)$ and $d\left( j \right)$ is as follows:

5 $$Sim2\left( {d\left( i \right),d\left( j \right)} \right) = \frac{{\mathop \sum \nolimits_{{t \in T_{d\left( i \right)} \cap T_{d\left( j \right)} }} \left( {D2_{d\left( i \right)} \left( t \right) + D2_{d\left( j \right)} \left( t \right)} \right)}}{{DV\left( {d\left( i \right)} \right) + DV\left( {d\left( j \right)} \right)}}$$

By formula (2), we can calculate $DV\left( {d\left( i \right)} \right)$ or $DV\left( {d\left( j \right)} \right)$ which is the semantic values of $d\left( i \right)$ or $d\left( j \right)$ similarly.$Sim2$ is another disease semantic similarity matrix and the element $Sim2\left( {d\left( i \right),d\left( j \right)} \right)$ is the semantic similarity of $d\left( i \right)$ and $d\left( j \right)$ according to disease semantic similarity model 2.

### Gaussian interaction profile kernel similarity for diseases

According to previous work, the Gaussian interaction distribution nuclear similarity of disease can be calculated [52]. We describe binary vector $V\left( {d\left( a \right)} \right)$ to stand for the interaction profiles of disease $d\left( a \right)$. The vector $IP\left( {d\left( a \right)} \right)$ is the *a*-*th* row vector of adjacency matrix A for the convenient utilization. The vector $IP\left( {d\left( b \right)} \right)$ is the *b*-*th* row vector of adjacency matrix A. We define the similarity between $d\left( a \right)$ and $d\left( b \right)$ as follow:

6 $$KD\left( {d\left( a \right),d\left( b \right)} \right) = exp\left( { - \gamma_{d} *\left\| {IP\left( {d\left( a \right)} \right) - IP\left( {d\left( b \right)} \right)} \right\|^{2} } \right)$$

where parameter $\gamma_{d}$ is applied to regulate the kernel bandwidth. It computes by normalizing original parameter $\gamma_{d} '$:

7 $$\gamma_{d} = \gamma_{d}^{'} /\left( {\frac{1}{nd}\mathop \sum \limits_{i = 1}^{nd} \left\| {IP\left( {d\left( i \right)} \right)} \right\|^{2} } \right)$$

### Gaussian interaction profile kernel similarity for miRNAs

The calculation process of the Gaussian profile kernel similarity for miRNAs is same as the process of diseases, and it can be described as follows:

8 $$KM\left( {m\left( a \right),m\left( b \right)} \right) = exp\left( { - \gamma_{m} *\left\| {IP\left( {m\left( a \right)} \right) - IP\left( {m\left( b \right)} \right)} \right\|^{2} } \right)$$

9 $$\gamma_{m} = \gamma_{m}^{'} /\left( {\frac{1}{nm}\mathop \sum \limits_{i = 1}^{nm} \left\| {IP\left( {m\left( i \right)} \right)} \right\|^{2} } \right)$$

where vector $IP\left( {m\left( a \right)} \right)$ is the *a*-*th* column vector of adjacency matrix *A* for the convenient utilization. The vector $IP\left( {m\left( b \right)} \right)$ is the *b*-*th* column vector of adjacency matrix *A*.

### Integrated similarity for diseases

An integrated disease similarity matrix SD is constructed [53]. The element $SD\left( {d\left( a \right),d\left( b \right)} \right)$ stand for gathered similarity between disease $d\left( a \right)$ and $d\left( b \right)$, and its formula is as follows:

10 $$SD\left( {d\left( a \right),d\left( b \right)} \right) = \left\{ {\begin{array}{*{20}l} {\frac{{Sim1\left( {d\left( a \right),d\left( b \right)} \right) + Sim2\left( {d\left( a \right),d\left( b \right)} \right)}}{2}} & \quad {if\, d\left( a \right),d\left( b \right) \,in\;Sim1\;and\;Sim2} \\ {KD\left( {d\left( a \right),d\left( b \right)} \right)} & \quad { others} \\ \end{array} } \right.$$

### Similarity for miRNAs

We use miRNA Gaussian interaction profile kernel similarity and miRNA functional similarity to construct miRNA similarity. Thus, the similarity between miRNA $m\left( a \right)$ and $m\left( b \right)$ is calculated as follows:

11 $$SM\left( {m\left( a \right),m\left( b \right)} \right) = \left\{ {\begin{array}{*{20}l} {FS\left( {m\left( a \right),m\left( b \right)} \right)} & {if\, m\left( a \right),m\left( b \right) \,in\;FS} \\ {KM\left( {m\left( a \right),m\left( b \right)} \right)} & {others} \\ \end{array} } \right.$$

### miRNAs sequence feature

Since miRNAs derive from distinct hairpin precursors (pre-miRNAs), we choose the sequences of pre-miRNAs to describe the sequence characteristics of miRNAs. More specifically, we first downloaded precursor sequences of 1057 miRNA needed from the miRBase. Secondly, we picked up sequence composition characters for miRNAs to obtain raw features. We pulled out 3-mer frequency for miRNA sequence (A, C, G, U), which is AAA, AAC … UUU [54]. And then we extract conjoint triad (3-mer) from miRNA sequences and get sequence feature matrixes as 64× (sequence-2) which represent the sequence information of each miRNA. After that, sequence feature matrixes are converted into new matrixes whose shape is 64 × 5 by Singular Value Decomposition (SVD) [55]. Hence, each miRNA sequence can be defined by a 320-dimensional vector according to reshape the sequence feature matrixes:

12 $$F_{seq} = \left( {f_{1} ,f_{2} ,f_{3} , \ldots f_{319} ,f_{320} } \right)$$

### Auto-encoder

**Fig. 6:**
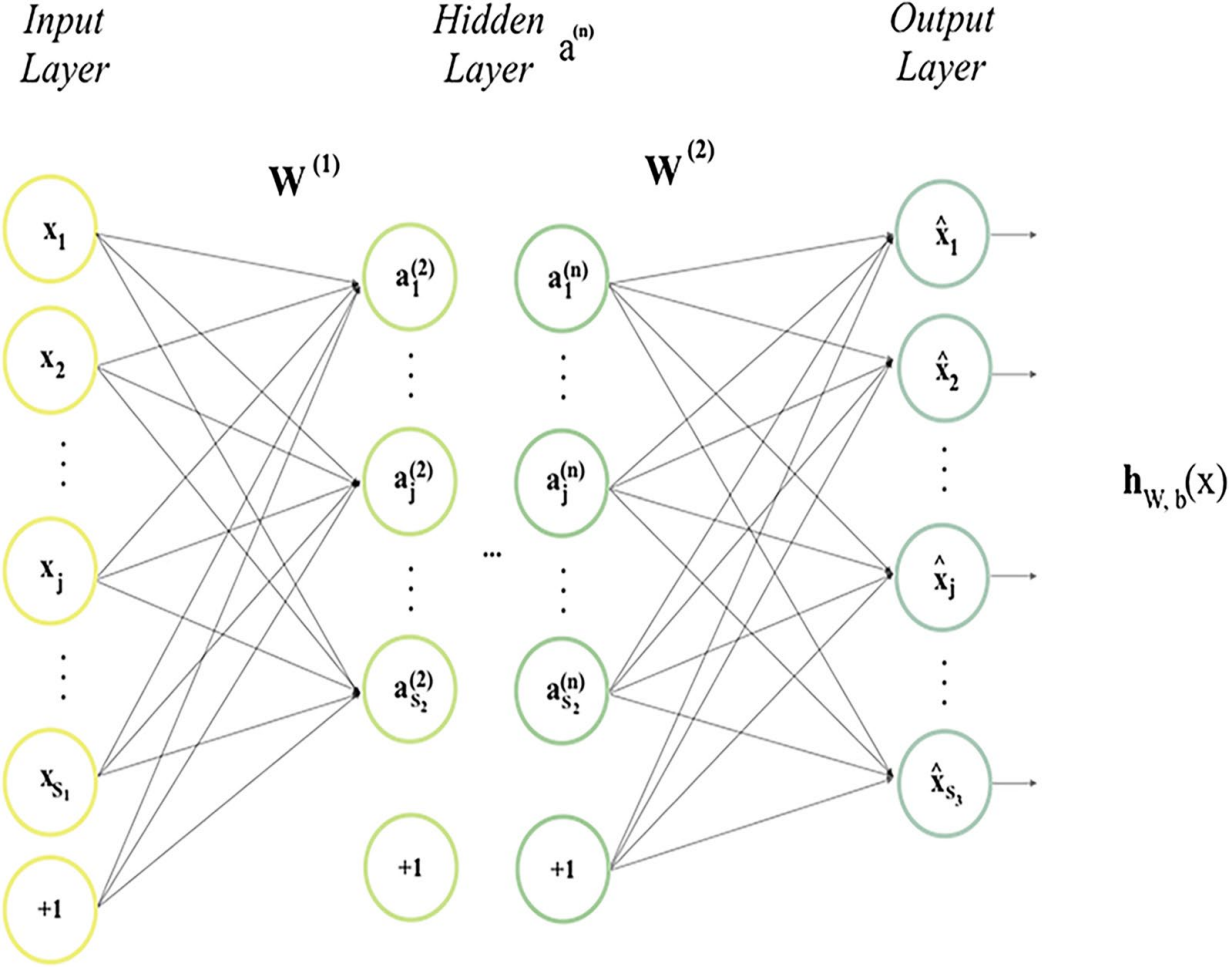
The structure of an auto-encoder model

Auto-encoder (AE) can avert the labor-intensive and feature designed by hand which is an unsupervised feature leaning methods. This method can conduct scientific experiments on computer vision, natural language process, audio processing and so on. The aim of AE is to make the input same as the output [56–58]. Substantially, AE is an unsupervised feed-forward neural network with the following structure (Fig. 6).

We choose $E = x^{\left( 1 \right)} ,x^{\left( 2 \right)} , \ldots ,x^{\left( n \right)} ,x^{\left( i \right)} \in R^{d}$ to be the unsupervised training examples. $a = \sigma \left( {W^{\left( 1 \right)} x + bias_{e} } \right)$ is the encoding function for mapping the input layer $x$ to hidden layer $a$ and $h = \sigma \left( {W^{\left( 2 \right)} a + bias_{d} } \right)$ is the decoding function for reconstituting $x$ from $a$. $W^{\left( 1 \right)}$ and $W^{\left( 2 \right)}$ are the relational parameters between two layers. $\sigma \left( x \right) = 1/\left( {1 + e^{ - x} } \right)$ is a non-linear mapping. $bias_{e}$ and $bias_{d}$ are vectors of bias parameters.

### MLMDA model

We describe a method named machine learning for miRNA–disease association prediction (MLMDA) based on machine learning. Functionally similar diseases are allied to similar miRNAs more likely, it is an assumption used to analyze data and also used in figuring target protein-drug association. There are four main steps of MLMDA: First, constructing positive set and negative set; second, combining miRNA and disease information matrixes to build feature vectors; third, reducing the number of feature’s dimensions; finally, constructing the forecast model to analyze potential miRNA–disease pairs. Next, we will discuss the details of each step.

Firstly, constructing positive set and negative set. We choose HMDD v3.0 as basic information and elected the confirmed miRNA–disease pairs as positive set. After that, we built negative set and it has three main process: (1) We chose a disease form all the 850 diseases; (2) We discretionarily choose one of the 1057 miRNAs; (3) A negative sample is constituted by the disease and the miRNA if the miRNA–disease association does not appear in the known miRNA–disease pairs. This process is repeated until we acquired negative samples.

Secondly, we constitute a miRNA–disease association as a feature vector and compute the Gaussian interaction profile kernel similarity, semantic similarity 1 and semantic similarity 2 between each disease. We define feature vector of disease $d\left( a \right)$ as follow:

13 $$SD\left( {d\left( a \right)} \right) = \left( {v_{1} ,v_{2} ,v_{3} , \ldots v_{849} ,v_{850} } \right)$$

where the $a$-th row vector of matrix $SD$ is defined as $SD\left( {d\left( a \right)} \right)$ and the combined similarity value of disease $d\left( a \right)$ and $d\left( b \right)$ is described as $v_{b}$.

We obtain miRNA similarity matrix through Gaussian interaction kernel profile similarity in the same way. $m\left( a \right)$ can be defined as follow:

14 $$SM\left( {m\left( a \right)} \right) = \left( {w_{1} ,w_{2} ,w_{3} , \ldots w_{1056} ,w_{1057} } \right)$$

where the $a$-th column vector of matrix $SM$ is described as $SM\left( {m\left( a \right)} \right)$. The combined similarity value between miRNAs is defined as $w_{b}$. Then, reducing $SM$ and $SD$ to 16 dimensions respectively. We can describe each miRNA–disease sample as a 32-dimensional vector according to combined disease similarity matrix and combined miRNA similarity matrix as follow:

15 $$F _{sim} = \left( {SD\left( {d\left( a \right)} \right),SM\left( {m\left( a \right)} \right)} \right)$$

where $F _{sim} = \left( {f_{1} ,f_{2} , \ldots ,f_{16} } \right)$, $\left( {f_{1} ,f_{2} , \ldots f_{16} } \right)$ represents the 16 combined similarity values of the disease and $\left( {f_{17} ,f_{18} , \ldots f_{32} } \right)$ is the 16 values of the miRNAs. After that, the sequence feature matrixes $F_{seq}$ are resized from 320 to 32 in same way. We can describe each miRNA–disease sample as a 64-dimensional vector based on combined resized $F _{sim}$ and combined resized $F_{seq}$ as follow:

16 $$F = \left( {{F_{sim}}^{\prime } ,{F_{seq}}^{\prime } } \right)$$

**Fig. 7:**
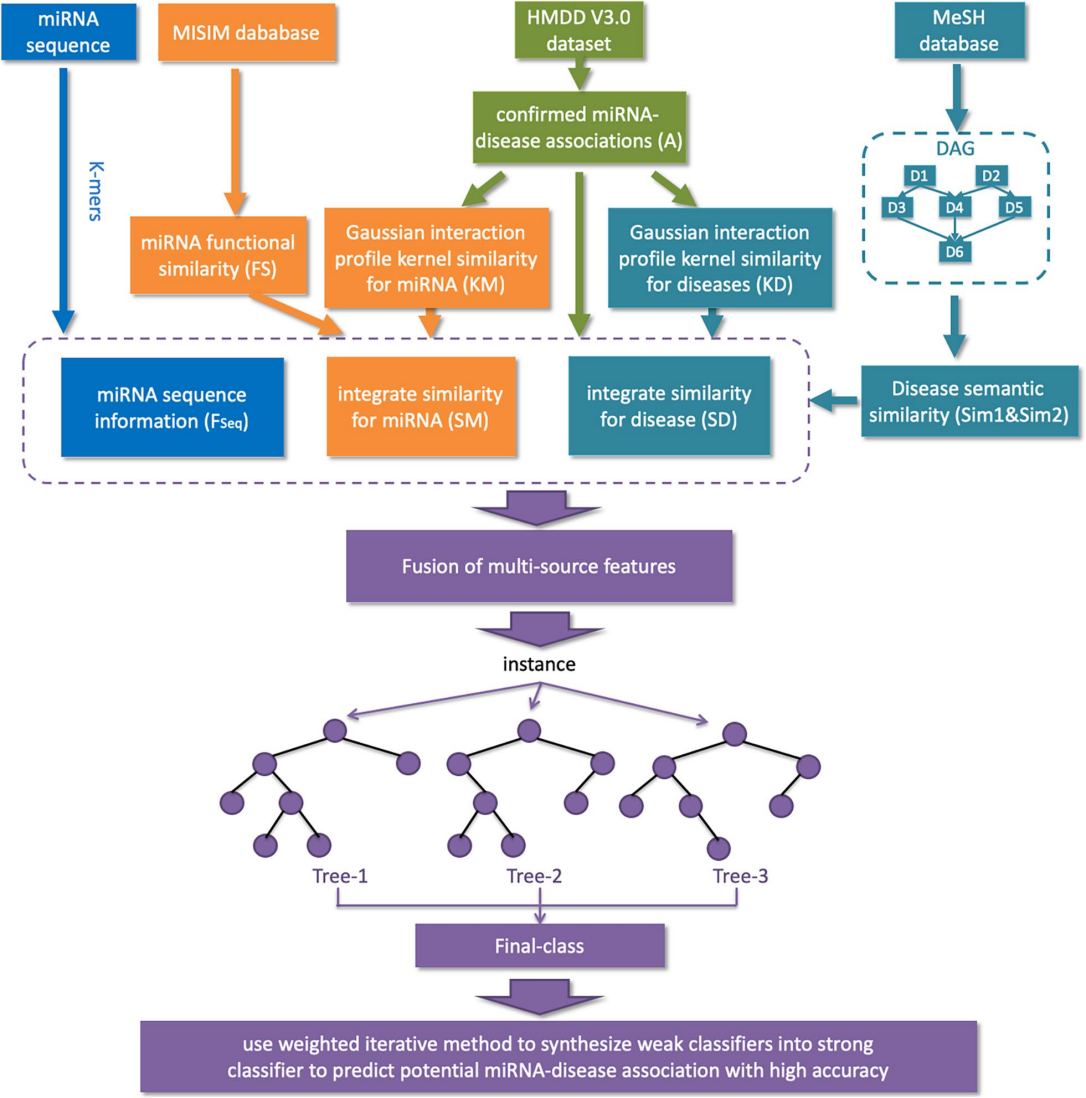
Flowchart of MLMDA model to predict unconfirmed miRNA–diseases associations

Finally, we use random forest classifier to build the prediction model. To be specific, the training sample is described as a 64-dimensional vector. We give a label of 1 if it is in the positive set and given a label of 0 if it is not in the negative set. And then, put the data of training samples into random forest classifier. After that, the model which can deduce potential miRNA–disease pairs can be gained. If the miRNA disease sample to be validated is higher, then the disease will be more likely to be associated with the miRNA (Fig. 7).

## Discussion

In this paper, functional similarities between miRNAs are quantified based on miRNA sequence information. The base of each nucleotide in the RNA is usually adenine (A), cytosine (C), guanine (G) or uracil (U). In general, the miRNA sequence may vary in length. To solve this problem, we first convert the sequence into a k-mer sparse matrix and then use the SVD alignment features. However, in previous experiments, we find that the traditional machine learning-based methods have huge feature vectors, and the data processing process is time consuming and resource intensive. So, we reduce the disease similarity information, miRNA similarity information and sequence information and use the combined feature vector, i.e., 64-D feature vector report result. We find that combining sequence information can successfully improve accuracy.

## Conclusion

The improvements of this method are effectively reducing the complexity of data processing while retaining most of the information of the feature and introducing the sequence information to improve the prediction accuracy. In comparison with other classifiers and other multi-source combination model, MLMDA have gained good performance. Besides, to further evaluate the prediction performance of MLMDA model, we have carried out case studies with three Human complex diseases including Lymphoma, Lung Neoplasm, and Esophageal Neoplasms. In this experiment MLMDA also have gained good performance. It is anticipated that the MLMDA model is a useful tool for the selection of miRNA biomarker candidates. In the future work, we will use more effective miRNA sequence information extraction method to build prediction models in the hope of achieving better results.

## Data Availability

The datasets analyzed during the current study are available from the corresponding author on reasonable request.

## Abbreviations

miRNA: microRNA
AE: auto-encoder
miRPD: protein-driven inference of miRNA–disease associations
HDMP: human disease-related miRNA prediction
HGIMDA: heterogeneous graph inference for miRNA–disease association prediction
RLSMDA: regularized least squares for miRNA–disease association
RKNNMDA: ranking-based KNN for miRNA–disease association prediction
MLMDA: the machine learning algorithm to predict miRNA–disease associations
SVM: support vector machine
DT: decision tree
HMDD: human microRNA disease database
MeSH: Medical Subject Headings
DAG: Directed Acyclic Graph
SVD: Singular Value Decomposition
RF: random forest classifier
